# High-fat diet feeding induces organ-specific vascular remodeling with distinct temporal dynamics in male mice

**DOI:** 10.1038/s42003-026-10092-4

**Published:** 2026-05-09

**Authors:** Emmi Pakarinen, Satu Paavonsalo, Madeleine H. Lackman, Yelin Subashi, Hanna M. Ruddock, Sinem Karaman

**Affiliations:** 1 Individualized Drug Therapy Research Program, Faculty of Medicine, University of Helsinki, Helsinki, Finland; 2 Wihuri Research Institute, Helsinki, Finland

**Keywords:** Mechanisms of disease, Animal disease models

## Abstract

Obesity-induced changes in adipose vasculature are well documented, but systematic analyses in other tissues have not been as extensive. Here, we analyze the vessel-covered area and endothelial cell (EC) numbers in seven non-adipose organs (liver, heart, intestine, kidney, lung, ear skin, retina) in male mice after short-term high-fat diet (HFD) feeding (5 days or 5 weeks), long-term HFD feeding (10 weeks), and weight loss (5 weeks of HFD followed by 5 weeks of chow feeding). We show HFD-induced morphological changes in the liver, heart, kidney, and intestinal vasculatures, and a negative correlation between body weight and vessel density in most of the analyzed tissues. Interestingly, changes in vessel area do not always reflect alterations in EC numbers. Additionally, both the intestine and ear skin show preserved vessel perfusion in response to obesity. This study provides a comprehensive analysis of how different HFD feeding durations affect organotypic vasculature and reports that HFD feeding induces organ-specific vascular remodeling with distinct temporal dynamics in mice.

## Abstract (web summary)

Analysis of vascular remodeling after various lengths of high-fat diet feeding reveals distinct organotypic responses in seven non-adipose tissues in male mice.

## Introduction

Blood vessels (BVs) lie between the circulation and the parenchymal cells. The vascular network is heterogenous, comprising of arteries, veins, and capillaries, all of which are lined by endothelial cells (ECs). ECs display both anatomical and functional heterogeneity, enabling them to provide support for the organs they inhabit^1^. Through the secretion of paracrine-acting factors, termed angiocrine factors, ECs communicate with the surrounding tissue, thereby contributing to the regulation of organ function (reviewed in ref. ^2^). This distinction of EC features in different organs is referred to as organ-specific or organotypic vasculature. A notable example of the organotypic characteristics of ECs is their response to altered signaling: Loss of vascular endothelial growth factor (VEGF) receptor signaling in ECs elicits diverse organotypic phenotypes—either regression or hyperproliferation of vessels—during both vascular development and maintenance in mice^3^. Thus, it is plausible that environmental changes may cause organ-specific responses in the vasculature.

Vascular dysfunctions are recognized in various diseases, including metabolic diseases. Obesity impairs metabolic homeostasis, which in turn impacts the vasculature. Even a single high-fat meal has been shown to transiently affect endothelial function by decreasing flow-mediated vasoactivity^4^. Lipids were shown to accumulate in ECs already 5 h after HFD feeding in mice^5^. Functionally, ECs have an active role in lipid metabolism and homeostasis as metabolic gatekeepers^6^. Capillary ECs in certain organs—including the intestine, adipose tissue, and muscle—use protein-facilitated transfer of fatty acids from systemic circulation into parenchymal cells^7^. Therefore, disturbances in metabolic homeostasis, such as an overload of circulating free fatty acids, can contribute to EC dysfunction^7^. The response of ECs to metabolic changes and obesity seems to also depend on the organ’s function. However, the timing of the organotypic vulnerability of ECs to obesity requires further understanding. Furthermore, the effect of weight loss after being overweight on organotypic vasculature and vessel density is understudied and requires more investigation.

Both subjects with obesity and animal models of obesity have been shown to have reduced BV density in the white adipose tissue compared to lean controls^8–12^. The reduced BV density, referred to as capillary rarefaction, is considered as one of the obesity-related vascular dysfunctions^13,14^. Capillary or microvascular rarefaction can be defined in multiple ways: a decrease in capillary density, a reduced number of capillaries in relation to parenchymal cells, or a loss of capillaries due to EC loss^13,15^. Furthermore, in obesity, tissue expansion due to enlargement of parenchymal cells (i.e., adipocyte hypertrophy in the adipose tissue or ectopic lipid accumulation in other tissues) can reduce BV density per unit area, despite the compensatory attempts of vessels to adapt to the tissue growth. In addition to morphological changes, obesity also causes hemodynamic changes^16^. During development, alterations in blood flow lead to a regression of low flow BV segments by ECs migrating away from these regions^17^. While anatomical rarefaction concerns the structural absence of capillaries, functional rarefaction can include, among other things, vessels that temporarily or permanently lack blood perfusion^18^. Thus, capillary rarefaction diminishes blood supply, leading to decreased oxygen and nutrient delivery^15^. Furthermore, angiogenic stimuli are reduced, and inflammation and the production of reactive oxygen species are increased^15^. In animal models, the degree of capillary rarefaction in different tissues during obesity remains poorly defined, largely depending on the obesity model, feeding duration, and diet type used, which complicates systematic comparisons of morphological changes and highlights the need for more comprehensive analyses.

In this study, we examined the effect of acute HFD feeding, HFD-induced obesity, and HFD withdrawal on organotypic vasculature. Vascular density and its changes in the adipose tissues have been extensively studied in various diet-induced overweight and obesity animal models as well as human subjects; however, corresponding information for other tissues remains incomplete. Here, we provide a comprehensive analysis of vessel morphology in selected non-adipose tissues (liver, heart, intestine, kidney, lung, ear skin, and retina), enabling direct comparison of vessel densities and EC percentages between tissues in different diet regimens.

## Results

### High-fat diet-induced obesity decreases podocalyxin-positive blood vessel area in the liver and heart

**Fig. 1:**
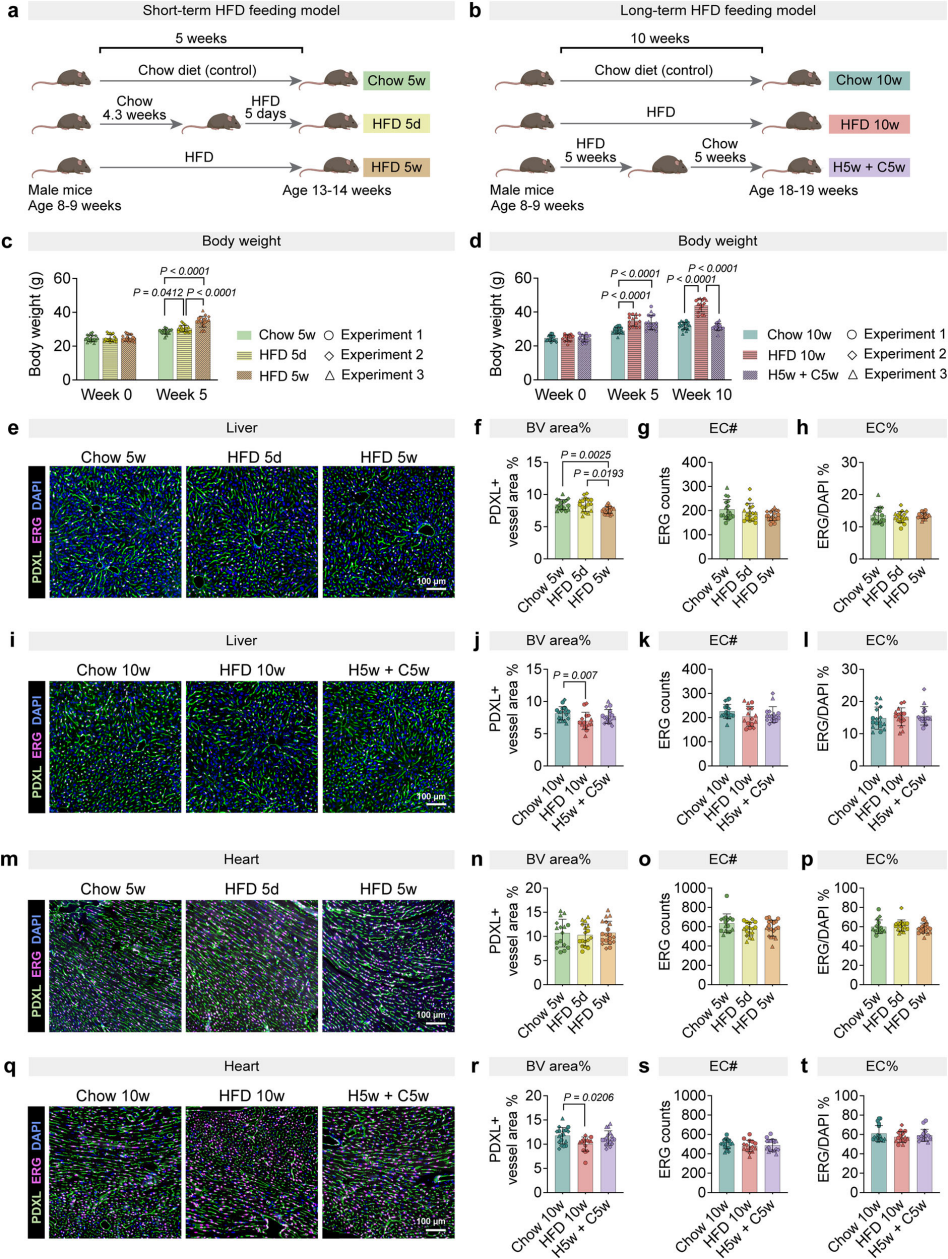
Blood vessel (BV) area% in the liver and heart decreases in response to obesity. Experimental designs of (**a**) the short-term high-fat diet (HFD) feeding model and **b** the long-term HFD feeding model. Body weights of mice measured (**c**) in the beginning and end of the short-term HFD feeding model and **d** in the beginning, midpoint, and end of the long-term HFD feeding model. **e** Representative images of BV (PDXL, green), endothelial cell (EC) nuclei (ERG, magenta), and cell nuclei (DAPI, blue) stainings of liver paraffin sections in the short-term HFD model and related quantifications for (**f**) BV area%, **g** EC#, and **h** EC%. **i** Representative images of BV (PDXL, green), EC nuclei (ERG, magenta), and cell nuclei (DAPI, blue) stainings of liver paraffin sections in the long-term HFD model and related quantifications for (**j**) BV area%, **k** EC#, and **l** EC%. **m** Representative images of BV (PDXL, green), EC nuclei (ERG, magenta), and cell nuclei (DAPI, blue) stainings of heart paraffin sections in the short-term HFD model and related quantifications for (**n**) BV area%, **o**) EC#, and **p**) EC%. **q** Representative images of BV (PDXL, green), EC nuclei (ERG, magenta), and cell nuclei (DAPI, blue) stainings of heart paraffin sections in the long-term HFD model and related quantifications for (**r**) BV area%, **s** EC#, and **t** EC%. The BV area% is expressed as the % of PDXL+ vessel area within the region of interest (ROI), the EC# is expressed as the number of ERG+ cells within the ROI, and the EC% is expressed as the % of ERG+ cells among DAPI+ cells. In the graphs, each dot represents one mouse (graphs **c**, **d**) or the mean value of one mouse (graphs **f**–**h**, **j**–**l**, **n**–**p**, **r**–**t**), and individual experiments are indicated by different shapes. The number of mice in the graphs, pooled from three independent experiments, are the following: Chow 5w (*n* = 17 for liver, *n* = 16 for heart), HFD 5d (*n* = 17), HFD 5w (*n* = 19), Chow 10w (*n* = 18, except graph l *n* = 17), HFD 10w (*n* = 15, except graph l *n* = 14), and H5w + C5w (*n* = 17). Error bars represent mean ± standard deviation. Statistical significance was determined using repeated measures two-way ANOVA followed by Tukey’s *post hoc* test for graphs **c** and **d**, one-way ANOVA with Welch’s correction followed by Tukey’s *post hoc* test for graph **f**, one-way ANOVA followed by Tukey’s *post hoc* test for graph **j**, and Kruskal-Wallis test followed by Dunn’s *post hoc* test for graph **r**. Graphs **a** and **b** partially created in BioRender. Paavonsalo, S. (2026) https://BioRender.com/3tmxkkw. BV blood vessel, C chow, EC endothelial cell, H/HFD high-fat diet, PDXL podocalyxin.

To model overweight and obesity, we used an HFD feeding strategy to induce weight gain and increase adiposity in mice. Since even a single day of HFD feeding was shown to change the whole-body metabolism in mice^19^, we designed two separate feeding regimens to investigate the effects of short-term and long-term HFD feeding. In the short-term feeding regimen, adult mice were fed with regular mouse chow for 5 weeks (Chow 5w) or with HFD for 5 days or 5 weeks (HFD 5d and HFD 5w, respectively, Fig. 1a) in an age-matched setup. In the long-term feeding regimen, mice were fed with either chow or HFD for 10 weeks (Chow 10w and HFD 10w, respectively, Fig. 1b). In addition, we included a third group where mice were first fed with HFD for 5 weeks, followed by chow feeding for another 5 weeks, modeling overweight followed by weight loss (H5w + C5w, Fig. 1b). This group is denoted as the weight loss group from here onwards. HFD feeding for 5 days, 5 weeks, and 10 weeks increased mouse body weights significantly compared to age-matched control mice fed with control chow diet (Chow 5w = 28.6 g, HFD 5d = 30.4 g, HFD 5w = 34.5 g; Chow 10w = 31.8 g, HFD 10w = 43.9 g, H5w + C5w = 31.2 g, Fig. 1c, d). Five days of HFD feeding resulted in a 6.3% higher final body weight compared to chow-fed mice, and 5 weeks of HFD feeding resulted in a 20.6% higher final body weight compared to chow-fed mice; while 10 weeks of HFD feeding resulted in a 38% higher final body weight compared to chow-fed mice. Mice in the weight loss group first gained weight during HFD feeding, but the weights became comparable to those of the control mice one to two weeks after changing the diet back to chow (final body weight difference: H5w + C5w vs. Chow 10w = –1.8%, Fig. 1d). We also measured the body composition of these mice to follow changes in fat and lean masses. In the short-term model, body fat percentages were significantly higher after 5 weeks of HFD feeding when compared to chow-fed mice and mice fed with HFD for 5 days (average body fat% (endpoint): Chow 5w = 13.9%, HFD 5d = 15.4%, HFD 5w = 23.1%, Supplementary Fig. 1a). Similarly, in the long-term model, body fat percentages initially increased significantly in response to 5 weeks of HFD feeding compared to chow-fed mice (average body fat% (5-week time point): Chow 10w = 15.1%, HFD 10w = 23.6%, H5w + C5w = 23.0%, Supplementary Fig. 1d). However, going back to chow diet feeding after 5 weeks of HFD reduced the fat percentages in the weight loss group to levels comparable to those of the chow-fed group (average body fat% (endpoint): Chow 10w = 17.3%, HFD 10w = 34.7%, H5w + C5w = 15.5%, Supplementary Fig. 1d). While the lean percentages seemed to decrease over time due to the prominent increase in body fat percentages (Supplementary Fig. 1b and 1e), the absolute lean mass showed a small but significant increase over time in both chow- and HFD-fed mice, consistent with normal murine growth (Supplementary Fig. 1c and 1f).

We next analyzed BV density and EC numbers, and calculated the EC percentages whenever DAPI staining was possible to quantify. Paraffin sections from liver samples were stained for podocalyxin (PDXL) for vessels, ERG for EC nuclei, and DAPI for all nuclei (Fig. 1e, i). Quantification of immunostainings revealed that the PDXL+ BV area% in the liver was not altered after 5 days of HFD feeding, but was significantly decreased (–9.5%) after 5 weeks of HFD feeding compared to both other groups in the short-term setting (average BV area%: Chow 5w = 8.4%, HFD 5d = 8.4%, HFD 5w = 7.6%, Table 1; Fig. 1f). Quantifications revealed that the absolute number of ECs was not significantly different between diet groups in the short-term setting (average EC#: Chow 5w = 205, HFD 5d = 194, HFD 5w = 178.3, Fig. 1g). We also wanted to analyze the percentage of ECs in different organs, and therefore, the number of ERG+ cells was normalized to the number of DAPI+ cells (ERG/DAPI%), referred to as the EC%. In the livers of chow-fed mice, ECs accounted for approximately 13% of cells (Table 2; Fig. 1h and 1l). The EC% did not change in response to HFD feeding in the short-term setting (Fig. 1h). In the long-term setting, BV coverage was decreased by 16.9% in mice fed with an HFD for 10 weeks compared with chow-fed mice (average BV area%: Chow 10w = 8.3%, HFD 10w = 6.9%, Table 1; Fig. 1j). Interestingly, the BV coverage after weight loss appeared similar to that observed after 5 weeks of HFD feeding (average BV area%: H5w + C5w = 7.3% vs. HFD 5w = 7.6%, Fig. 1j). Because these groups were not age-matched (weight loss group is 5 weeks older), a formal statistical comparison was not performed. Moreover, the EC# and EC% remained similar between diet groups in the long-term setting (Table 2; Fig. 1k, l).

**Table 1:** Quantification of the blood vessel (BV) area% in different tissues of mice fed with chow or high-fat diet (HFD)

	Blood vessel area% ± SD (n=group size)					
	Short-term HFD experiment			Long-term HFD experiment		
Tissue (Marker)	Chow 5w	HFD 5d	HFD 5w	Chow 10w	HFD 10w	HFD 5w + Chow 5w
Liver (PDXL)	8.4 ± 0.8 (*n* = 17)	8.4 ± 1.0 (*n* = 17)	7.6 ± 0.5 (*n* = 19)	8.3 ± 1.0 (*n* = 18)	6.9 ± 1.6 (*n* = 15)	7.3 ± 1.2 (*n* = 17)
Heart (PDXL)	10.7 ± 2.9 (*n* = 16)	10.3 ± 2.3 (*n* = 17)	10.7 ± 2.4 (*n* = 19)	11.8 ± 1.7 (*n* = 18)	10.0 ± 1.5 (*n* = 15)	11.3 ± 1.5 (*n* = 17)
Kidney (EMCN)	10.8 ± 1.9 (*n* = 17)	10.1 ± 1.6 (*n* = 17)	9.7 ± 2.0 (*n* = 19)	9.5 ± 1.6 (*n* = 18)	8.9 ± 1.9 (*n* = 15)	9.5 ± 1.0 (*n* = 17)
Lung (PDXL)	26.5 ± 3.5 (*n* = 17)	27.1 ± 3.5 (*n* = 17)	28.9 ± 4.7 (*n* = 19)	23.1 ± 4.2 (*n* = 18)	24.8 ± 3.5 (*n* = 15)	23.7 ± 5.0 (*n* = 17)
Intestinal villus (PDXL)	54.1 ± 1.1 (*n* = 17)	53.8 ± 0.8 (*n* = 17)	53.5 ± 0.9 (*n* = 19)	52.4 ± 1.2 (*n* = 18)	52.7 ± 1.7 (*n* = 15)	52.4 ± 2.1 (*n* = 17)
Ear skin (PDXL)	19.0 ± 1.5 (*n* = 17)	18.6 ± 1.2 (*n* = 17)	18.0 ± 3.0 (*n* = 19)	16.2 ± 1.9 (*n* = 18)	16.2 ± 1.5 (*n* = 15)	16.2 ± 1.3 (*n* = 17)
Retina deep plexus (iB4)	17.7 ± 0.8 (*n* = 9)	17.5 ± 0.7 (*n* = 11)	17.9 ± 0.9 (*n* = 11)	17.4 ± 1.4 (*n* = 18)	17.6 ± 1.2 (*n* = 15)	17.3 ± 1.2 (*n* = 17)
Retina superficial plexus (iB4)	13.1 ± 1.2 (*n* = 9)	13.1 ± 1.5 (*n* = 11)	13.0 ± 1.3 (*n* = 11)	12.5 ± 0.8 (*n* = 18)	12.5 ± 0.8 (*n* = 15)	12.2 ± 1.1 (*n* = 17)

Average values of the BV area% in the liver, heart, kidney, lung, intestine, ear skin, and retina (deep and superficial plexus) collected from mice that were fed either with chow or HFD. In each group, mice were pooled from three independent experiments and the number of mice in each analysis is mentioned in brackets.

*BV* blood vessel, *EMCN* endomucin, *HFD* high-fat diet, *iB4* isolectin B4, *PDXL* podocalyxin, *SD* standard deviation.

**Table 2:** Percentages of endothelial cells (ECs) in various tissues collected from mice fed with chow or high-fat diet (HFD)

	ERG/DAPI% ± SD (n=group size)					
	Short-term HFD experiment			Long-term HFD experiment		
Tissue	Chow 5w	HFD 5d	HFD 5w	Chow 10w	HFD 10w	HFD 5w + Chow 5w
Liver	13.6 ± 2.4 (*n* = 17)	13.1 ± 1.7 (*n* = 17)	13.4 ± 0.9 (*n* = 19)	13.0 ± 2.7 (*n* = 17)	13.7 ± 3.0 (*n* = 14)	13.3 ± 2.3 (*n* = 17)
Heart	59.9 ± 7.1 (*n* = 16)	61.1 ± 5.9 (*n* = 17)	58.6 ± 5.2 (*n* = 19)	61.0 ± 8.3 (*n* = 18)	57.4 ± 5.7 (*n* = 15)	59.2 ± 6.2 (*n* = 17)
Kidney	18.7 ± 3.2 (*n* = 17)	16.3 ± 2.2 (*n* = 17)	16.5 ± 2.2 (*n* = 19)	17.2 ± 2.0 (*n* = 18)	16.0 ± 2.0 (*n* = 15)	16.7 ± 2.0 (*n* = 17)
Lung	56.5 ± 6.5 (*n* = 17)	57.6 ± 5.0 (*n* = 17)	55.4 ± 4.5 (*n* = 19)	51.6 ± 3.3 (*n* = 18)	50.4 ± 4.0 (*n* = 15)	53.1 ± 4.7 (*n* = 17)

The percentage of ERG+ EC nuclei among DAPI+ cell nuclei was quantified in the liver, heart, kidney, and lung tissues that originated from mice that had been fed with chow or HFD for different lengths. In each group, mice were pooled from three independent experiments and the number of mice in each analysis is mentioned in brackets.

*HFD* high-fat diet, *SD* standard deviation.

To ensure that the reduction in BV density is not due to the downregulation of PDXL expression in the ECs, we additionally analyzed another endothelial marker, vascular endothelial (VE)-Cadherin, by Western blotting the liver samples from one 10-week cohort (Supplementary Fig. 2a). When we quantified the relative band intensities, we could detect a similar reduction in VE-Cadherin expression in the total liver lysates after 10 weeks of HFD feeding, suggesting that the reduced PDXL+ BV area% is due to reduced vascular content and not due to a selective downregulation of PDXL expression (Supplementary Fig. 2a and 2b).

Next, we analyzed the heart vasculature by staining paraffin sections (Fig. 1m, q). Exposure to HFD feeding for 5 days or 5 weeks did not alter the PDXL+ BV area%, which was approximately 10% in the heart, when compared with chow-fed mice in the short-term setting (Table 1; Fig. 1n). Moreover, the EC# and EC% were similar between the study groups in the short-term setting (Table 2; Fig. 1o, p). However, 10 weeks of HFD feeding resulted in a 15.3% decrease in the PDXL+ BV area% in the heart compared to chow-fed mice (Table 1; Fig. 1r). Despite the decreased BV area% in the heart, neither the EC# nor EC% was significantly decreased in the long-term setting (Table 2; Fig. 1s, t). Thus, immunohistochemical analyses revealed changes in the vascular coverage of the liver and heart in response to HFD feeding, although the EC# or EC% were not altered in these tissues.

### High-fat diet feeding alters the endothelial cell number without changing the vessel area in the small intestine and kidney

**Fig. 2:**
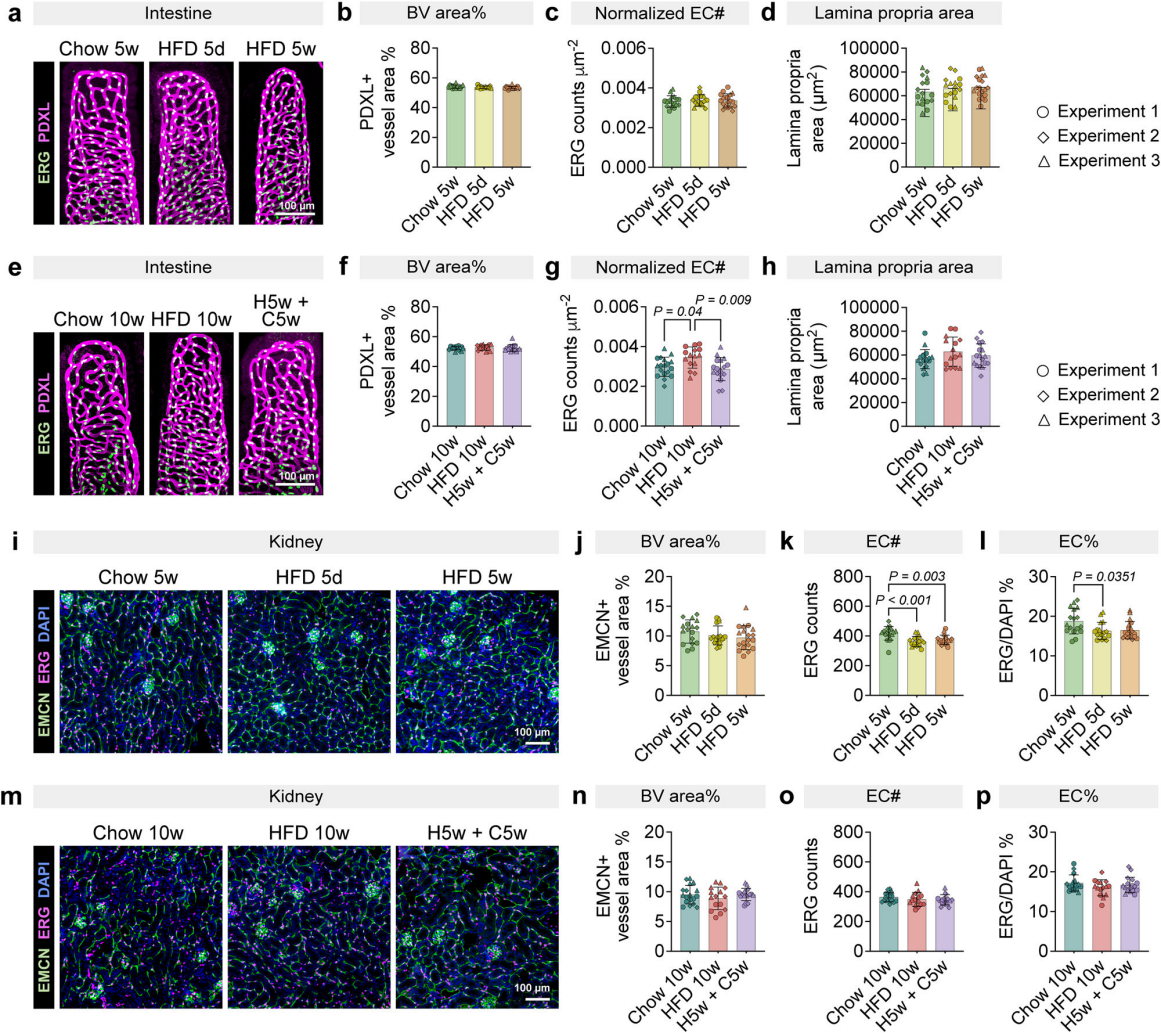
The number of endothelial cells (ECs) is altered in the intestine and kidney in response to high-fat diet (HFD) feeding. **a** Representative images of blood vessel (BV, PDXL, magenta) and EC nuclei (ERG, green) stainings of intestine whole-mounts in the short-term HFD model and related quantifications for (**b**) BV area%, **c** normalized EC#, and **d** lamina propria area. **e** Representative images of BV (PDXL, magenta) and EC nuclei (ERG, green) stainings of intestine whole-mounts in the long-term HFD model and related quantifications for (**f**) BV area%, **g** normalized EC#, and **h** lamina propria area. For the intestine, the BV area% is expressed as the % of PDXL+ vessel area within the lamina propria area and the normalized EC# is expressed as the number of ERG+ cells within the lamina propria area. **i** Representative images of BV (EMCN, green), EC nuclei (ERG, magenta), and cell nuclei (DAPI, blue) stainings of kidney paraffin sections in the short-term HFD model and related quantifications for (**j**) BV area%, **k** EC#, and **l** EC%. **m** Representative images of BV (EMCN, green), EC nuclei (ERG, magenta), and cell nuclei (DAPI, blue) stainings of kidney paraffin sections in the long-term HFD model and related quantifications for (**n**) BV area%, **o** EC#, and **p** EC%. For the kidney, the BV area% is expressed as the % of EMCN+ vessel area within the region of interest (ROI), the EC# is expressed as the number of ERG+ cells within the ROI, and the EC% is expressed as the % of ERG+ cells among DAPI+ cells. In the graphs, each dot represents the mean value of one mouse, and individual experiments are indicated by different shapes. The number of mice in the graphs, pooled from three independent experiments, are the following: Chow 5w (*n* = 17), HFD 5d (*n* = 17), HFD 5w (*n* = 19), Chow 10w (*n* = 18), HFD 10w (*n* = 15), and H5w + C5w (*n* = 17). Error bars represent mean ± standard deviation. Statistical significance was determined using one-way ANOVA followed by Tukey’s *post hoc* test for graphs **g** and **k**, and Kruskal-Wallis test followed by Dunn’s *post hoc* test for graph (**l**). BV blood vessel, C chow, EC endothelial cell, EMCN endomucin, H/HFD high-fat diet, PDXL podocalyxin.

Next, we analyzed the vasculature in the intestine, which, in addition to its well-established roles, is considered one of the major endocrine tissue that secretes essential hormones involved in metabolism^20^. To visualize the whole blood vasculature in the villus, we performed whole-mount stainings on the proximal jejunum of the small intestine (Fig. 2a, e). We found that the BV area% and EC# normalized to the lamina propria area, as well as the lamina propria area remained similar to control mice after 5 days and 5 weeks of HFD feeding (Table 1; Fig. 2b–d). Similarly, neither longer HFD feeding nor weight loss affected the area of BVs and lamina propria in intestinal villi (Table 1**;** Fig. 2f, h). Instead, the EC# normalized to the lamina propria area was significantly increased (15.6%) in obese mice in the long-term HFD setting (Fig. 2g).

The increased number of ECs in the intestinal villi suggested a possibility for increased EC proliferation in response to 10 weeks of HFD feeding. To address this, we stained intestinal samples from an additional cohort for the proliferation marker Ki67 in addition to ERG (Supplementary Fig. 3a). First, we counted ECs in the villi and found higher ERG+ EC counts normalized to the lamina propria area in obese mice (Supplementary Fig. 3b), which was in line with the first three experiments (Fig. 2g). However, the percentage of Ki67+/ERG+ double- positive ECs in the villus remained low (< 0.4%), and there were no differences in the proliferating EC% between the diet groups (Supplementary Fig. 3c). Whether there were differences in the proliferation of ECs in the crypt regions could not be quantified from whole-mount samples due to the high proliferation signal in the crypt.

Next, we analyzed the kidney, which, similar to the heart, is highly vascularized. To assess the BV area% in the kidney, we stained paraffin sections with an antibody recognizing endomucin (EMCN), as PDXL is highly expressed in kidney podocytes in addition to vascular endothelia^21^ (Fig. 2i, m). Despite the unaltered BV area% (Table 1**;** Fig. 2j), the absolute number of ECs per region of interest (ROI) was reduced after 5 days (–13.2%) and 5 weeks (–10.5%) of HFD feeding as compared to chow-fed mice (Fig. 2k). In agreement with this, the EC% was also decreased after 5 days and 5 weeks of HFD feeding (Table 2; Fig. 2l), although the difference between chow and HFD fed mice at the 5-week time point was not significant. Mice fed with HFD for 10 weeks and the weight loss mice showed similar BV area%, EC#, and EC% as the chow-fed control mice (Tables 1 and 2; Fig. 2n–p).

In the kidney, both the EC# and EC% decreased after 5 days and 5 weeks of HFD feeding when compared to chow-fed mice. Therefore, we wanted to assess the ratio of cells undergoing apoptosis in the kidney sections to see whether the decrease in ECs was caused by apoptosis. Due to problems with high background as reported earlier, the TUNEL assay did not work for the kidney sections^22^. Instead, we then examined apoptotic DNA fragmentation  by staining kidney sections for phosphorylated histone 2 AX (p-H2AX). Sections from different diet groups had a similar, low number of positive cells which were not located on the vessels (Supplementary Fig. 3d). Thus, we did not observe DNA fragmentation in the ECs, which could have been one explanation for the reduced EC counts after short-term HFD feeding.

### Up to 10 weeks of high-fat diet feeding does not alter blood vessel coverage in the lung, ear skin, or retina

**Fig. 3:**
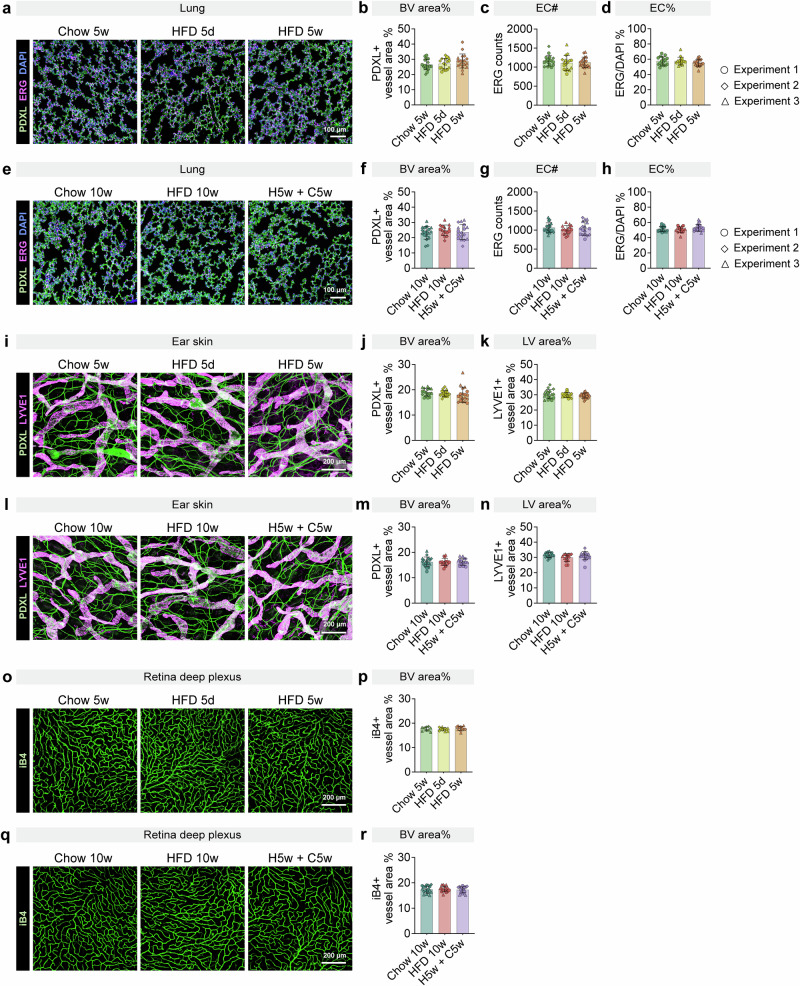
High-fat diet (HFD) feeding does not affect blood vessel (BV) area in the lung, ear skin, or retina. **a** Representative images of BV (PDXL, green), endothelial cell (EC) nuclei (ERG, magenta), and cell nuclei (DAPI, blue) stainings of lung paraffin sections in the short-term HFD model and related quantifications for (**b**) BV area%, **c** EC#, and **d** EC%. **e** Representative images of BV (PDXL, green), EC nuclei (ERG, magenta), and cell nuclei (DAPI, blue) stainings of lung paraffin sections in the long-term HFD model and related quantifications for (**f**) BV area%, **g** EC#, and **h** EC%. For the lung, the BV area% is expressed as the % of PDXL+ vessel area within the region of interest (ROI), the EC# is expressed as the number of ERG+ cells within the ROI, and the EC% is expressed as the % of ERG+ cells among DAPI+ cells. **i** Representative images of BV (PDXL, green) and lymphatic vessel (LV, LYVE1, magenta) stainings of ear skin whole-mounts in the short-term HFD model and related quantifications for (**j**) BV area% and **k** LV area%. **l** Representative images of BV (PDXL, green) and LV (LYVE1, magenta) stainings of ear skin whole-mounts in the long-term HFD model and related quantifications for (**m**) BV area% and **n** LV area%. For the ear skin, the BV area% is expressed as the % of PDXL+ BV area within the ROI and the LV area% is expressed as the % of LYVE1+ vessel area within the ROI. **o** Representative images of BV (iB4, green) stainings of retinal deep plexus whole-mounts in the short-term HFD model and related quantifications for (**p**) BV area%. **q** Representative images of BV (iB4, green) stainings of retinal deep plexus whole-mounts in the long-term HFD model and related quantifications for (**r**) BV area%. For the retina, the BV area% is expressed as the % of iB4+ vessel area within the ROI. In the graphs, each dot represents the mean value of one mouse, and individual experiments are indicated by different shapes. The number of mice in the graphs, pooled from three independent experiments, are the following: Chow 5w (*n* = 17), HFD 5d (*n* = 17), HFD 5w (*n* = 19), Chow 10w (*n* = 18), HFD 10w (*n* = 15), and H5w + C5w (*n* = 17). As an exception, short-term HFD model retina sample numbers were the following: Chow 5w (*n* = 9), HFD 5d (*n* = 11), and HFD 5w (*n* = 11). Error bars represent mean ± standard deviation. BV blood vessel, C chow, EC endothelial cell, H/HFD high-fat diet, iB4 isolectin B4, LV lymphatic vessel, LYVE1 lymphatic vessel endothelial hyaluronan receptor 1, PDXL podocalyxin.

Next, we stained lung paraffin sections for PDXL to visualize the dense vasculature (Fig. 3a, e). Quantification of the BV coverage in the lung sections demonstrated that the BV area% did not change after short-term HFD feeding (Table 1; Fig. 3b). In addition, the EC# was similar and the EC% remained similar at approximately 56% between diet groups (Table 2; Fig. 3c, d). Similarly, in the long-term experiments, there were no differences between the diet groups in these morphological parameters (Tables 1 and 2; Fig. 3f–h).

In the ear skin, HFD feeding for 5 days or 5 weeks did not result in significant changes in the BV area% compared to chow-fed mice (Table 1; Fig. 3i, j). In addition to the BV area%, we quantified the lymphatic vessel (LV) area% in the ear skin with a staining against lymphatic vessel endothelial hyaluronan receptor 1 (LYVE1; Fig. 3i, l). Quantification indicated that there were no differences in the LV area% between diet groups in the short-term model (average LV area%: 30%, Fig. 3k). Similarly, mice with 10-week-long HFD feeding or weight loss presented similar BV area% (all averaged 16.2%) as their corresponding control mice (Table 1; Fig. 3l, m), and the LV area% were unaltered (Fig. 3n).

To assess whether HFD feeding affects the BV area% in the retina, we stained retinal whole-mount samples for *Griffonia simplicifolia* lectin I isolectin B4 (iB4), which efficiently binds to retinal ECs. We first determined the BV area% in the deep plexus (Fig. 3o, q). The BV area% in the deep plexus was approximately 17.5% in all diet groups in both short-term and long-term experiments (Table 1; Fig. 3p, r). We also examined the superficial plexus of the retina. Images analyzed from the superficial plexus were mainly acquired around the arteries (Supplementary Fig. 4a and 4c). In the superficial plexus, quantification showed no differences in the BV area% between the experimental groups (Table 1; Supplementary Fig. 4b and 4d).

### Vessel perfusion measured by a 10-min lectin accessibility assay in the intestine and ear skin is not altered in diet-induced obesity

**Fig. 4:**
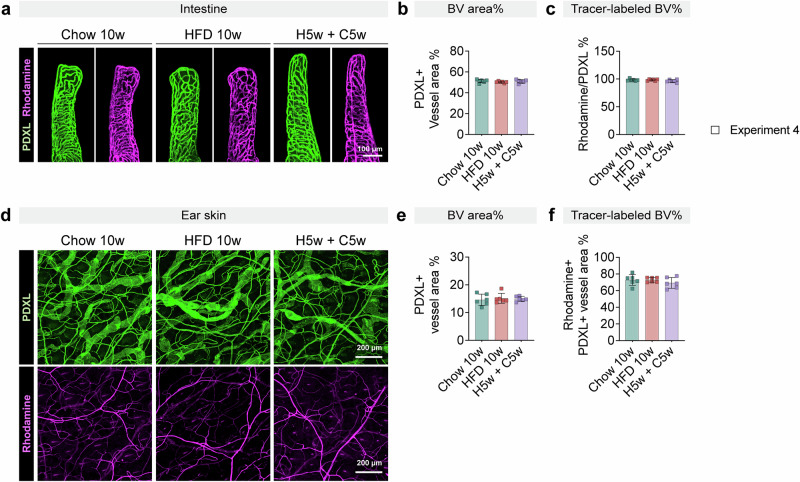
Vessel perfusion shown by lectin-labeling is not altered in the intestine or ear skin upon high-fat diet (HFD)-induced obesity. **a** Representative images of blood vessel (BV) staining (PDXL, green) and rhodamine tracer signal (magenta) of intestine whole-mounts in the long-term HFD model and related quantifications for (**b**) BV area% and **c** tracer-labeled BV area%. For the intestine, BV area% is expressed as % of PDXL+ vessel area within lamina propria area and tracer-labeled BV area% is expressed as % of rhodamine+ area relative to PDXL+ area. **d** Representative images of BV staining (PDXL, green) and rhodamine tracer signal (magenta) of ear skin whole-mounts in the long-term HFD model and related quantifications for (**e**) BV area% and **f** tracer-labeled BV area%. For the ear skin, the BV area% is expressed as the % of PDXL+ vessel area within the region of interest (ROI) and the tracer-labeled BV area% is expressed as the % of rhodamine+/PDXL+ double-positive vessel area within the PDXL+ BV area. In the graphs, each dot represents the mean value of one mouse. Number of mice per group, *n* = 6. Error bars represent mean ± standard deviation. BV blood vessel, C chow, H/HFD high-fat diet, PDXL podocalyxin.

While vessels can stay anatomically and morphologically intact, their functional integrity or capacity to access blood circulation might be impaired in pathological conditions. Therefore, we additionally sought to investigate BV perfusion to complement our morphological assessments. To detect potential differences in the capacity of BVs to access blood circulation in obese mice within a 10-min experimental window, we repeated the long-term HFD feeding protocol and injected the mice with rhodamine-labeled lectin to assess vessel perfusion status. Importantly, when referring to vessel perfusion in this study, we particularly mean the vasculature that is open to blood circulation and accessible to lectin binding. In line with our initial three experiments (Fig. 1d; Supplementary Fig. 1d), HFD feeding for 10 weeks resulted in significantly increased mouse body weights and body fat%, and mice in the weight loss group initially gained weight but their body weights decreased to levels comparable to chow-fed mice after HFD withdrawal (Supplementary Fig. 5a and 5b). To visualize the BV perfusion status after HFD feeding, we intravenously injected fluorescent rhodamine-lectin, which binds to the glycocalyx of the BVs upon contact, and harvested tissues after 10 min. BVs were also stained for PDXL to determine the vessels anatomically. First, we analyzed intestinal whole-mount samples, where the quantified PDXL+ BV area% (Fig. 4a, b) was in line with the results of our previous experiments (Fig. 2f). Next, the percentage of rhodamine-lectin+ perfused vessels in relation to the PDXL+ vessels were quantified from intestinal images. We found that nearly all BVs in the villus vasculature were labeled with rhodamine-lectin, and the percentage of labeling was similar across the diet groups (Fig. 4c). Thus, neither HFD-induced obesity nor weight loss by HFD withdrawal altered vessel perfusion levels within a 10-min experimental window in the intestinal villus vasculature.

We also analyzed BV perfusion in the ear skin of the same mice by staining whole-mount samples from the ear for PDXL and calculating the percentage of the rhodamine+/PDXL+ BV area within the PDXL+ BV area (Fig. 4d). The BV area% did not change between diet groups (Fig. 4e), which was in line with our previous experiments (Fig. 3m). The rhodamine+ signal was measured within the PDXL+ BV areas, accounting for an approximate 70% rhodamine signal coverage in control mice (Fig. 4f). This value did not change in obese mice or weight loss mice within the 10-min test (Fig. 4f). Similar to the intestine, obesity and weight loss did not change vessel perfusion levels in the ear skin in our 10-min-long perfusion test.

### Correlation analysis reveals associations among vascular parameters within organs, across organs, and with body weight

To gain further insights into how the BV area%, EC%, and physiological parameters such as body weight and fat mass might correlate within individual mice, we next performed paired correlation analyses. Pearson correlation revealed statistically significant associations in three main comparison categories: (i) between the BV area% and EC% within individual tissues (within-organ correlations), (ii) between vascular parameters (BV area% and/or EC%) and physiological measures (body weight and fat mass), and (iii) between the BV area% and EC% across different tissues (across-organ correlations).

**Fig. 5:**
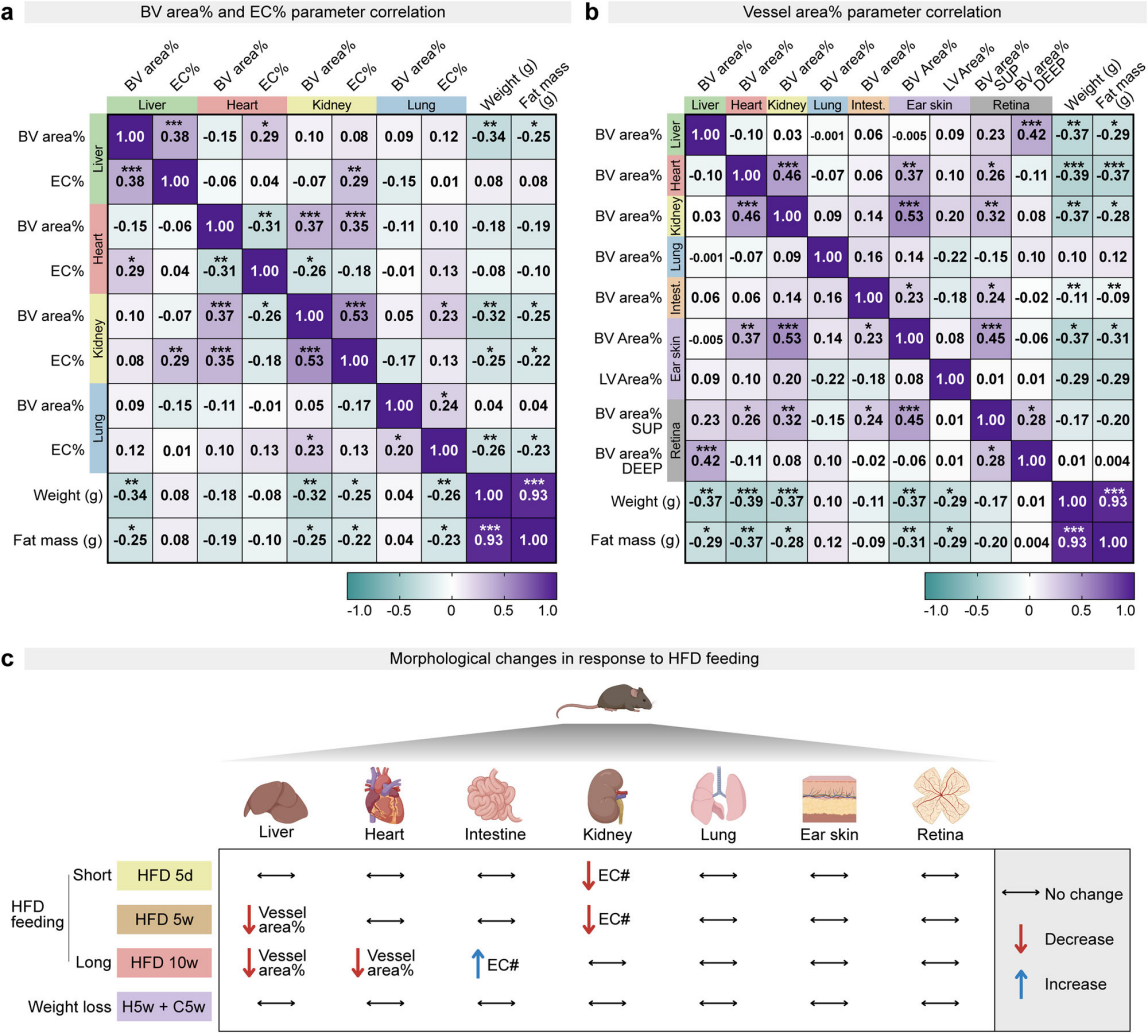
Correlation and grouped analyses showing organ-specific vascular remodeling in high-fat diet (HFD) feeding and weight loss. Pearson correlation matrices of (**a**) vascular parameters (blood vessel (BV) area% and endothelial cell (EC)%) and physiological parameters (body weight and fat mass) across liver, heart, kidney, and lung; and **b** vascular parameters (BV area% and lymphatic vessel (LV) area%) and physiological parameters (body weight and fat mass) across liver, heart, kidney, lung, intestine, ear skin, and retina. The heatmaps show Pearson correlation coefficients (r) across individual mice (*n* = 100 in graph **a**, *n* = 77 in graph **b**), and the color key indicates r values. The BV area% corresponds to the podocalyxin (PDXL)+ BV area% in the liver, heart, lung, intestine, and ear skin; the endomucin (EMCN)+ BV area% in the kidney; and the isolectin B4 (iB4)+ BV area% in the retina. The LV area% in the ear skin represents the lymphatic vessel endothelial hyaluronan receptor 1 (LYVE1)+ LV area%. Statistically significant correlations are indicated as follows: * = *P* < 0.05, ** = *P* < 0.01, *** = *P* < 0.001. **c** Schematic summary of the main morphological changes in the BV area% and EC# in the liver, heart, intestine, kidney, lung, ear skin, and retina after HFD-feeding for short-term (HFD 5d and 5w) and long-term (HFD 10w) and weight loss (H5w + C5w) cohorts in comparison to age-matched, chow-fed control mice. Downward pointing red arrows indicate a statistically significant decrease in the BV area% or EC#, upward pointing blue arrows indicate a statistically significant increase in the BV area% or EC#, and double-headed horizontal black arrows indicate no statistically significant difference in the BV area% or EC#, all relative to age-matched, chow-fed control mice. Graph **c **partially created in BioRender. Paavonsalo, S. (2026) https://BioRender.com/3m1f5gj. BV blood vessel, C chow, DEEP deep plexus, EC endothelial cell, H/HFD high-fat diet, LV lymphatic vessel, PDXL podocalyxin, SUP superficial plexus.

First, considering within-organ correlations, we found the strongest and most consistent positive correlations between the BV area% and EC% in the liver, kidney, and lung (Fig. 5a). On the other hand, there was a significant negative correlation between these two parameters in the heart (Fig. 5a). These findings suggest that the BV area% and EC% can vary independently and that these parameters should be measured separately because a change in the BV area% does not necessarily seem to reflect a change in the EC% or vice versa.

Second, we found significant negative correlations between vascular (BV area% and EC%) and physiological (body weight and fat mass) parameters in most tissues (Fig. 5a). This indicates that increased body weight and adiposity are associated with reduced vascular density across the analyzed organs. Notably, in addition to the heart and liver which showed significant differences in the grouped analyses, the skin and kidney also exhibited significant negative correlations between vessel density and body weight and fat mass parameters (Fig. 5b). These findings suggest that vessel density closely correlates with body weight in these tissues, although the grouped analyses did not reveal statistically significant differences. Interestingly, the lung, intestine, and retina showed no correlations between vascular and physiological parameters, suggesting that vascular density in these tissues is likely independent of body weight and adiposity (Fig. 5b).

Third, when analyzing across-organ correlations of the BV area% (Fig. 5b), we found that the heart BV area% positively correlated with the BV area% of the kidney, ear skin, and the superficial layer of the retina. Similarly, the ear skin BV area% positively correlated with the BV area% in the heart, kidney, intestine, and the superficial layer of the retina (Fig. 5b). The lung showed weak or no associations with other tissues, suggesting a distinct vascular response (or a lack of it) to dietary alterations (Fig. 5b). The EC% showed the strongest positive across-organ correlations between (i) liver and kidney, and (ii) liver and heart, whereas the EC% negatively correlated between the heart and the kidney (Fig. 5a).

Finally, to facilitate integration of the grouped and correlation-based analyses, we included a graphical summary highlighting the key vascular changes observed across organs (Fig. 5c).

## Discussion

Obesity is associated with vascular perturbations, including microvascular rarefaction that concerns not only adipose tissue but also various other tissues. Since previous studies have focused on examining the vasculature of individual tissues at specific stages of obesity, there has been a need for more systematic studies assessing obesity-induced morphological changes across different tissues over time. Thus, we sought to investigate how varying durations of HFD feeding and weight loss affect vessel morphology in seven non-adipose tissues (liver, heart, intestine, kidney, lung, ear skin, and retina) from a large cohort of male mice and identified tissues that are more sensitive or more resistant to HFD-induced vascular remodeling. Moreover, we examined whether vessel perfusion is affected in selected tissues in long-term HFD feeding. We also performed paired correlation analyses on this large mouse cohort and identified across-organ correlations among the BV area%, EC%, body weight, and fat mass. Our collective results suggest that HFD feeding induces organ-specific vascular remodeling with distinct temporal dynamics in mice, warranting further mechanistic investigations.

Among the studied organs, the liver appears to be highly sensitive to HFD feeding, as we found that the PDXL+ BV area% decreased by 9.5% after 5 weeks and by 16.9% after 10 weeks of HFD feeding. This reduction in the PDXL+ area% is likely due to structural vascular remodeling rather than downregulation of the PDXL marker, as our Western blot analysis showed significant downregulation of VE-Cadherin, another vascular marker, in total liver lysates after 10 weeks of HFD feeding. Beyond the BV area%, we also calculated the EC% in the liver and found it to be approximately 13%, which is in line with previous reports showing that liver sinusoidal ECs comprise 15-20% of the cells in the liver^23^. The decreased liver BV density observed in our study at 5 and 10 weeks of HFD feeding could be related to hepatic lipid accumulation rather than inflammation. This is because liver steatosis has been reported as early as after 4 days of HFD feeding^24^, whereas inflammatory gene expression has been shown to increase only after 16 weeks of HFD feeding^25^. Hepatic lipid accumulation may reduce vessel density through morphological remodeling of BVs/ECs, as it has been reported to reduce liver EC porosity and fenestrae^26^. However, the increased lipid content in the liver alone does not explain the decreased BV area%, because it has also been shown that BV density may remain unchanged despite a significant increase in triglycerides in the liver^27^. Furthermore, our results demonstrate that HFD withdrawal and consequent weight loss resulted in BV area% remaining similar to the 5-week HFD levels, indicating no reversal toward the 5-week chow levels. Notably, the BV area% in the weight loss group was also not statistically different from that of age-matched 10-week chow-fed mice, consistent with observations of vascular density decreasing with age^28^. Altogether, these findings indicate that HFD-induced capillary rarefaction in the liver can occur as early as after 5 weeks of HFD feeding, potentially initially driven by lipid overload, with minimal reversal following weight loss.

In addition to the liver, the heart vasculature was also affected by HFD feeding, but only at 10 weeks, causing a 15% decrease in the PDXL+ BV area% in the heart. Our result aligns with previous studies, with one demonstrating that 14 weeks of HFD feeding causes a 10% decrease in the CD31+ vessel area of the heart^29^, and another study reporting a lower capillary density in the left ventricular tissue biopsies of human subjects with obesity compared to lean subjects^30^. In addition to the BV area% analyses, we quantified the cardiac EC% to be 60%, which is consistent with a previous report presenting that ECs constitute 55.0 ± 6.0% of cardiac nuclei^31^. While we observed a decrease in the BV area%, we found no changes in the EC# or EC% in the heart after 10 weeks of HFD feeding. In fact, our paired correlation analysis showed a significant negative correlation between the BV area% and EC% in the heart, indicating that these parameters are not interchangeable and should be assessed separately, at least in the heart. Altogether, based on our and others’ results, it seems that obesity-induced reduction in vessel density in the heart requires a longer time to develop than in the liver.

The intestine is a highly responsive organ to metabolic changes since already a single day of HFD feeding is reported to increase the proliferation of intestinal epithelial cells^19^. In general, HFD feeding increases the number of intestinal crypt-based stem cells and their regenerative capacity^32,33^. However, studies have primarily focused on the impact on the mucus and epithelial barriers, while ECs have received comparatively less attention^34^. Here, we found no changes in the BV area%, lamina propria area, or lectin binding in the small intestine villi, but detected a 15.6% increase in the number of intestinal ECs after 10 weeks of HFD feeding. Surprisingly, despite the increased EC#, we did not detect differences in the expression of the proliferation marker Ki67 in the intestinal villi in any region at the 10-week time point, although hypoxia, for example, has been shown to induce detectable EC proliferation in the tip of the villus^35^. It is possible that changes in proliferation occur between 5 and 10 weeks of HFD feeding, and, therefore, we were unable to capture such changes in the proliferating EC% at the experimental endpoint. Dynamics of EC changes and proliferation should be studied in more depth in the future. Notably, since we did not detect increases in the BV area%, this may indicate that the EC size decreases, or that the vessel diameter is reduced upon HFD feeding, resulting in an overall unchanged BV area%. Altogether, our results suggest that while HFD-induced obesity causes an increased number of intestinal ECs, this remodeling does not impact the intestinal BV area% within the 10-week-long study time frame.

We did not observe obesity-induced changes in the BV area% in the kidney, but the number of ERG+ ECs was decreased by 13.2% after 5 days and by 10.5% after 5 weeks of HFD feeding. In line with our finding on decreased EC counts, transcriptionally, kidney ECs are reported to be vulnerable to obesity and that these changes are the least reversible upon weight loss when compared to the liver, heart, and many other tissues^36^. Since lipids have been shown to accumulate in the kidneys of HFD-fed mice^37,38^, it is possible that this lipid overload could cause acute downregulation of ERG expression in the kidney. While we did not observe BV area% changes in the kidney, other studies have reported increased glomerular capillary density and decreased peritubular capillary density upon obesity^13^. For example, glomerular hypertrophy and enhanced capillarization have been reported in the kidney during the early stages of obesity^39^. Another study showed that renal vessels in rats proliferate during obesity progression and vessel density increases only later in obesity^40^, whereas peritubular capillary density has been shown to decrease in mice after 12 weeks of HFD feeding, measured with CD31 and CD34 markers^41,42^. The fact that we did not observe changes in the kidney BV area% in our study might be because we analyzed the kidney BV area% as a whole, instead of analyzing glomerular and peritubular capillaries separately. All in all, our results suggest that HFD feeding as short as 5 days or 5 weeks decreases the number of renal ECs, but up to 10 weeks of HFD feeding does not impact the total BV area% in the analyzed kidney regions or potential changes in renal vascular density require a longer HFD feeding duration.

While the vasculature in some tissues appears to be significantly affected by HFD feeding, we found that the vasculature in other tissues might not be as profoundly affected by HFD feeding within the time frames we studied. For instance, we did not observe HFD-induced changes in the BV area% in the lung, ear skin, or retina. For the lung, our findings are consistent with another study showing that the total lung vessel area is not decreased in individuals with obesity compared to lean controls^43^. In contrast, a rodent study reported increased vessel areas in the lung in both HFD-induced and genetically obese rats^44^. In the ear skin, we did not observe changes in the BV area% although overweight and obesity have been linked to reduced dermal capillary density and impaired capillary recruitment. For example, Altintas et al. report lower dermal capillary density in overweight patients compared to lean subjects^45^, and De Ciuceis et al. report similar reductions in both normotensive and hypertensive patients with obesity^46^. While we did not observe vascular density changes in the grouped analyses, we found a significant negative correlation between vascular density in the ear skin and body weight, suggesting that body weight and adiposity have an impact on the dermal vasculature in mice. As for the perfusion of the ear skin vessels, one study reported unaffected vessel perfusion in the skin after 20 weeks of HFD feeding^47^, while another study observed that particularly long HFD feeding of 32 weeks decreased spontaneous capillary perfusion in the ear skin in obese rats compared to control rats^48^. Based on these results, it is not surprising that up to 10 weeks of HFD feeding did not cause changes in the BV area% or vessel perfusion level in the skin of mice in our study settings. For the retina, our findings of the unaltered BV area% in the superficial and deep capillary plexuses comply with previous studies. For example, unaltered capillary vessel densities have been reported in the intermediate and deep plexuses of genetically obese mice^49^, and changes in vascular densities among individuals with obesity were observed only in the optic nerve head, but not in the superficial or deep capillary plexuses^50^. Despite the lack of morphological alterations, it has been shown that obesity causes oxidative stress and inflammation in the retina^51^, suggesting that retinal BV densities might be affected only by HFD feeding longer than the 10 weeks used in this study. Alternatively, obesity-induced effects might be detectable only in the intermediate capillary plexus which was not examined here. Taken together, our results suggest that HFD feeding up to 10 weeks does not cause obesity-induced changes in the BV area% in the lung, ear skin, and retina, and that potential vascular changes may require a longer time to develop.

To summarize our findings from the grouped analyses, we observed HFD-induced changes in the BV area% only in the liver and heart, while vascular densities of the intestine, kidney, lung, ear skin, and retina remained unaltered within 10 weeks of HFD feeding. At the transcriptomic level, Bondareva et al. also identified alterations in ECs of the liver and heart, in addition to other tissues, in response to diet-induced obesity^36^. They describe organs that are major hubs for lipid metabolism to be most vulnerable to diet-induced obesity at the gene expression level in diet-induced obesity^36^. Their multi-organ EC single-cell RNA sequencing dataset enabled comparison of our morphological findings with differentially expressed genes (DEGs) found in capillary/sinusoidal ECs of the liver, heart, lung, and kidney in their dataset. In the liver, where we observed a decrease in BV density during HFD feeding, we could identify a set of DEGs in the Bondareva et al. dataset that may be related to vascular remodeling^36^. In heart capillary ECs, we observed mainly downregulated genes upon Western diet-induced obesity, for example, related to cell and system development, possibly suggesting a change in EC state. Interestingly, there seemed to be an increase in the number of DEGs with longer feeding periods (4 or 6 months compared to 3 months), underlining that, for some organs, it might take longer than our 10-week feeding period to observe vascular changes also at the morphological scale. All in all, based on DEGs alone, we could not directly link reduced vascular density to changes in gene expression in these mouse models of diet-induced obesity and weight loss. However, since the liver and heart have a high basal metabolic rate, i.e. resting energy expenditure^52^, our data together with Bondareva et al. led us to speculate that tissues with a high basal metabolic rate (among the analyzed tissues) are particularly susceptible to vascular alterations upon obesity.

In addition to our grouped analyses, Pearson correlation analyses across our large mouse cohort revealed relationships between vascular parameters, body weight, and fat mass that were not apparent from the group-wise comparisons alone. The identification of such correlations in tissues without significant grouped differences highlights how correlation-based approaches can capture more gradual and weight-dependent relationships between vascular remodeling and adiposity. Together, these findings suggest that paired correlation analyses provide complementary insight into tissue-specific vascular responses to obesity.

Our study complements prior research that lacked systematic morphological studies of non-adipose tissues by providing a comprehensive analysis of several such tissues from a large cohort of mice across varying HFD feeding durations. Nevertheless, we recognize that our study also has limitations. First, using HFD feeding to induce obesity resulted in variation between mouse weights, especially in the long-term cohorts, as reported in previous literature^53,54^. We also observed some variation in weight gain among chow-fed mice, which might be explained by differences in the control diet across experiments. In the future, control mice could be fed a diet with fewer calories from fat (e.g., 10%) and with similar sucrose levels in both the control and high-fat diet. Second, we were unable to quantify the EC# and EC% across all tissues, and we did not analyze parenchymal cell numbers or additional vessel parameters including branching, diameter, and pericyte coverage. Normalizing the BV area to the number of parenchymal cells can be used to identify anatomical vessel rarefaction in tissues where obesity causes parenchymal cell hypertrophy or ectopic lipid accumulation. Third, due to technical difficulties, we could analyze BV perfusion measured by lectin labeling in only two tissues. It should also be kept in mind that in vivo lectin labeling reports the accessibility of open vessels to circulating blood during the tracer circulation time window and does not capture dynamic perfusion parameters such as blood flow velocity, vasoreactivity, or longer-term functional rarefaction. Fourth, since female and male mice exhibit sex-specific differences in the angiogenic responses in adipose tissue during obesity^55^, the effects of sex on the vascular densities in other tissues should also be investigated. Finally, while the diet-induced obesity model used in this study effectively replicates the metabolic and physiological effects of excessive dietary fat intake observed in humans, complementary models should be employed to better capture the multifactorial nature of the disease because human obesity arises from both excessive caloric intake and high-fat consumption, in addition to genetic factors.

To conclude, our study provides a systematic overview of how diet-induced overweight, obesity, and weight loss impact vascular morphology and how vascular and physiological parameters correlate in various non-adipose tissues. Our findings raise important open mechanistic questions regarding tissue-specific vascular remodeling that remain to be addressed in future studies. For instance, it would be interesting to explore whether different EC populations (e.g., capillary and venous ECs) within the same organ differ in their vulnerability to HFD feeding. Furthermore, it would be important to determine whether the mechanisms of vessel regression in pathological conditions like obesity are similar to those during developmental processes, such as EC migration during vessel remodeling^56^. Previous studies have linked capillary rarefaction in aging to reduced angiogenic stimuli^28^. Thus, defining the potential contribution of altered angiogenic signaling in cardiometabolic diseases represents an important and interesting direction for future studies. Moreover, mechanistic and functional studies (e.g., flow speed measurements) should be performed with tissues where capillary rarefaction is more pronounced than in the tissues examined in this study (e.g., adipose tissues) or in other experimental settings (e.g., after longer HFD duration).

## Methods

### Mice

All experiments were performed with male mice of the inbred C57BL/6JRj strain (Janvier Labs) starting at the age of 8–9 weeks. Adult male mice were used as models to study obesity, as they display more consistent weight gain under HFD conditions compared to females, whose responses vary and depend on thermoneutral housing^57^. Mice were maintained in a pathogen-free facility with controlled temperature, a 12 h light/dark cycle, and *ad libitum* access to water and food. Mice were monitored daily for skin condition, mobility, behavior, and basic physiological functions, and weighed weekly. Cages were changed frequently to ensure hygiene, and welfare was further supported through environmental enrichment and social housing whenever possible. We set the following predetermined humane endpoints: If abnormalities appear, mice will receive supportive care and be closely monitored for 1–2 days; if no improvement occurs, they will be euthanized. Mice will also be euthanized if their body weight decreases by ≥15% within one week or ≥20% over a longer period, unless they are in the weight loss group. However, no mice underwent euthanization before the end of the experiments. Terminal anesthesia with a lethal dose of ketamine and xylazine was used to ensure humane death. All animal experiments were approved by the committee for Animal Experiments of the District of Southern Finland (ESAVI/22335/2019 and ESAVI/25789/2022). We have complied with all relevant ethical regulations for animal use. Other tissues from these mice were also utilized in other studies to maximize data and minimize total mouse use in accordance with 3R principles.

### Study design and high-fat diet feeding

In this study, two separate settings were used to investigate how different durations of HFD feeding affect organotypic vasculature. In the short-term feeding regimen, mice were fed either chow or HFD for 5 weeks (Chow 5w and HFD 5w, respectively). Five days before the end of the experiment, a group of chow-fed mice was switched to HFD feeding for the remaining five days to provide age-matched mice with a short HFD feeding duration (HFD 5d). In the long-term feeding regimen, mice were placed on chow or HFD for a total of 10 weeks (Chow 10w and HFD 10w, respectively). Additionally, one group of HFD-fed mice was placed back on a chow diet for 5 weeks after 5 weeks of HFD feeding, creating a model of HFD withdrawal and weight loss (H5w + C5w). In both regimens, the feeding of HFD for 5 and 10 weeks was started for mice at the age of 8–9 weeks. The HFD included 60% of calories from fat (D12492i, Research diets). The chow-fed groups received two different chow diets: Teklad 2918 and 2916 with 18% and 12% calories from fat, respectively, due to the revision of mouse facility standards. The use of two different chow diets created a potential confounder. To model obesity, we established a pre-determined criterion for the 10-week HFD feeding period: Only mice whose final body weight was at least 10% higher than the average body weight of chow-fed controls after 10 weeks were included in the HFD 10w group. Mice that did not fulfill the criterion were excluded as they did not meet the criteria for overweight or obesity. At the end of the experiment, mice were euthanized using a combination of xylazine (20 mg/kg, Bayer, 6047) and ketamine (150 mg/kg, Intervet International, 511485), followed by thoracic cavity exposure and cardiac blood collection.

### Statistics and reproducibility

The experimental unit in this study is a single mouse. No statistical methods were used to pre-determine sample size; however, group sizes were chosen based on previous experience with HFD models. The sample size of 5 was chosen as the minimum required to perform statistical tests. Prior to the start of the study, mice were weighed and assessed for body composition, then assigned to experimental groups to ensure balanced baseline characteristics. Experiments were performed in three independent replicates, and data were pooled to demonstrate reproducibility. An exception to this is the lectin perfusion experiment, which was conducted only once. The number of mice per group were: Chow 5w = 17, HFD 5d = 17, HFD 5w = 19, Chow 10w = 18, HFD 10w = 17 (of which 2 were excluded), H5w + C5w = 17. In the perfusion experiment, mouse numbers per group were: Chow 10w = 6, HFD 10w = 6, H5w + C5w = 6. The number of mice in short-term experiments were: Experiment 1 = 20, Experiment 2 = 18, Experiment 3 = 15, in the long-term experiments: Experiment 1 = 16, Experiment 2 = 18 (of which 2 were excluded), Experiment 3 = 18; and in the perfusion experiment = 18. In total, 123 mice were used in the study, of which 2 were excluded as these mice did not meet our overweight criterion. Blinding was not performed during the experiments or tissue collection, but the researchers were blinded to mouse group allocation during imaging and data analysis when group identity was not evident from tissue morphology.

All results are expressed as the mean ± standard deviation (SD) and considered to be significant when *P* < 0.05. Statistical tests for significant differences are indicated in figure legends. The continuous data collected were assumed to be normally distributed, and distribution was confirmed with a Shapiro-Wilk test. For data with a normal distribution, equality of variances was tested by a Brown-Forsythe test. Next, a one-way analysis of variance (ANOVA) was used for comparing three groups, followed by a *post hoc* test of Tukey’s multiple comparisons test if the ANOVA was significant. In case the variances were not equal, Welch’s correction for ANOVA was applied to assess the differences between the groups. In case data points were not normally distributed, a non-parametric Kruskal-Wallis test was applied to compare three groups, followed by Dunn’s multiple comparisons test. Pearson correlation analyses were conducted to measure linear correlations between (i) vascular parameters (BV area% and EC%) and physiological parameters (body weight and fat mass) across liver, heart, kidney, and lung from 100 mice; and (ii) vascular parameters (BV area% and LV area%) and physiological parameters (body weight and fat mass) across liver, heart, kidney, lung, intestine, ear skin, and retina from 77 mice. The Pearson correlation analyses were conducted using paired data from individual mice, missing values were excluded from the analyses, and correlation coefficients (r) and corresponding two-tailed *P* values were reported. All statistical analyses and graph preparations were performed with GraphPad Prism software (versions 10.4.0 and 10.6.1).

### Body composition analysis

The body composition of experimental mice was measured using Minispec (Bruker) and analyzed by using Minispec Plus software (version 7.0.0.). The measurements were conducted at the beginning and at the end of the experiment. Furthermore, in the long-term HFD experiments, the body composition of mice was analyzed at the midpoint – before switching the weight loss group to chow diet.

### Whole-mount stainings

Harvested tissues were fixed with 4% paraformaldehyde (PFA) in phosphate-buffered saline (PBS, pH 7.4) for one day. Intestine samples were stained according to the protocol described earlier^58^. Briefly, intestine and ear skin samples were permeabilized with PBS containing 0.3% Triton-X 100 (PBS-Tx). Donkey immunomix (DIM) containing 5% normal donkey serum (Biowest, S2170), 0.2% bovine serum albumin (BSA, Biowest, P6154), 0.05% sodium azide, and 0.3% PBS-Tx was used for blocking. Samples were incubated with primary antibodies diluted in DIM from 16 h up to three days at +4°C on a shaker. Primary antibodies used were goat anti-PDXL (1:400, R&D Systems, AF1556, RRID: AB_354858), rabbit anti-ERG (1:1000, Abcam, ab92513, RRID: AB_2630401), rabbit anti-LYVE1 (1:1000, AngioBio, 11-034, RRID: AB_2813732), and rat anti-Ki67 (1:500, Invitrogen, 14-5698-82, RRID: AB_10854564). Samples were washed with 0.3% PBS-Tx for several hours, followed by incubation with secondary antibodies diluted in PBS at least overnight at +4 °C. Secondary antibodies used were: donkey anti-goat Alexa Fluor Plus 488 (1:500, Invitrogen, A32814, RRID: AB_2762838), donkey anti-rabbit Alexa Fluor Plus 647 (1:500, Invitrogen, A32795, RRID: AB_2762835), and donkey anti-rat Alexa Fluor 488 (1:500, Invitrogen, A21208, RRID: AB_2535794). Samples were washed with 0.3% PBS-Tx, post-fixed with 1% PFA for 10 min at room temperature, and washed with PBS before mounting on slides with Mowiol mounting medium (Sigma-Aldrich, 81381) (preparation described in ref. ^58^).

Retinas were dissected and incubated in Pblec buffer (0.1 mM CaCl_2_, 0.1 mM MgCl_2_, 0.1 mM MnCl_2_, and 1% Triton-X 100 in PBS, pH 6.8), followed by overnight incubation with biotinylated *Griffonia simplicifolia* lectin I isolectin B4 (1:25, Vector Laboratories, B-1205, RRID: AB_2314661) diluted in Pblec buffer. After washing for a minimum of 6 h with PBS, the retinas were incubated with a fluorescent streptavidin conjugate (Streptavidin Alexa Fluor 488 conjugate, Invitrogen, S11223) diluted in PBS overnight. On the following day, the retinas were washed with PBS, post-fixed with 1% PFA for 10 min at room temperature, washed again, and flat-mounted with Mowiol mounting medium by making four radial incisions to create a four-petaled configuration.

### Immunofluorescence staining of paraffin sections

Tissues were fixed in 4% PFA for one day, followed by tissue processing and paraffin embedding. Paraffin blocks were then cut into 5 μm-thick sections. After paraffin removal, sections were boiled in alkaline Tris-EDTA buffer (10 mM Tris, 1 mM EDTA, 0.5% Tween-20, pH 9.0) for antigen retrieval. After cooling down, the sections were washed twice with PBS and blocked with a blocking solution composed of 0.2% BSA (Biowest, P6154) and 2.5% normal donkey serum (Biowest, S2170) diluted in PBS. The sections were incubated in primary antibodies diluted in the blocking solution overnight at +4°C. Primary antibodies used were goat anti-PDXL (1:400, R&D Systems, AF1556, RRID: AB_354858), rat anti-EMCN (1:500, Santa Cruz Biotechnology, sc-65495, RRID: AB_2100037), rabbit anti-ERG (1:1000, Abcam, ab92513, RRID: AB_2630401), and rabbit anti-p-H2AX (Ser139) (1:400, Cell Signaling Technology, 9718, RRID: AB_2118009). Next day, sections were washed with Tris-NaCl-Tween20 (TNT) buffer (150 mM NaCl, 100 mM Tris, 0.05% Tween20). Sections were incubated with secondary antibodies and DAPI (1:1000, Tocris, 5748) diluted in 0.05% PBS-Tween for 1 h. Secondary antibodies used were donkey anti-goat Alexa Fluor Plus 488 (1:500, Invitrogen, A32814, RRID: AB_2762838), donkey anti-rat Alexa 594 (1:500, Invitrogen, A21209, RRID: AB_2535795), and donkey anti-rabbit Alexa Fluor Plus 647 (1:500, Invitrogen, A32795, RRID: AB_2762835). After washes with TNT and PBS buffers, the sections were fixed with 1% PFA for 5 min. Washes with PBS and mQ-H_2_O preceded mounting with Prolong Gold (Invitrogen, P36930) mounting reagent.

### Imaging of stained whole-mount tissues and paraffin sections

All whole-mount samples, including the intestine, retina, and ear skin, were imaged with an LSM 780 confocal microscope (ZEISS) equipped with 10x Plan-Apochromat with numerical aperture (NA) 0.45 and 20x Plan-Apochromat with NA 0.80 air objectives through Hamamatsu Orca Flash 4.0 LT camera and Zen Pro 2.3 software. For intestine and ear skin samples, confocal Z-stacks were captured to include the full thickness of the vessel network. Stainings on paraffin tissue sections were imaged with AxioImager (ZEISS) equipped with 20x Plan-Apochromat with NA 0.80 air objectives through Hamamatsu Orca Flash 4.0 LT camera and Zen Pro 2.3 software. Images shown were prepared using Adobe Photoshop (version CC 2024) and Adobe Illustrator (version CC 2024) software, and the brightness of images was adjusted in the figures for visualization.

### Image analysis

Vessel area%, EC#, and EC% were quantified from the acquired images using the ImageJ software (ImageJ 1.52 h; National Institutes of Health). Depending on the analyzed tissue, the BV area% was quantified as a percentage of PDXL+ (liver, heart, intestine, lung, ear skin), EMCN+ (kidney), or iB4+ (retina) BV area within the area of a ROI. PDXL is a glycoprotein expressed on the inner surface of vessels and in kidney podocytes^21^, which is why we used EMCN instead of PDXL for kidney vessel quantifications. To accompany the blood vessel area% measurements, we also determined the number of ERG+ EC nuclei in the same ROIs. ERG+ and DAPI+ cell counts were quantified as a number of nuclei within the ROI. The EC% was quantified as a percentage of ERG+ cells among DAPI+ cells. For the perfusion analyses, the tracer-labeled BV area% was quantified either as a percentage of rhodamine+ area relative to the PDXL+ BV area (intestine) or as a percentage of the rhodamine+/PDXL+ BV area within the PDXL+ BV area (ear skin). Each dot in the graphs represents the average of these values obtained from multiple images per individual mouse.

For the liver, kidney, lung, and heart, ROIs of size 665.6 μm x 665.6 μm were used. More specifically, for the livers, four randomly selected ROIs containing central veins were imaged from the left hepatic lobe. For the kidneys, four ROIs were collected from renal cortical regions enriched in glomeruli, proximal to the outer cortex. For the lungs, four ROIs were acquired from the peripheral parenchymal areas of the left upper lung lobe, carefully excluding major airways and large vessels. For the hearts, three ROIs were selected from the outer myocardial wall of the right ventricle. Similarly, EC# and EC% were quantified from the same ROIs.

For the intestinal analysis, three ROIs approximately 4 cm distal to the pyloric region of the stomach (jejunum) were acquired for each mouse. The area of lamina propria in each villus was defined individually. The area of histologically detectable PDXL+ capillaries within the lamina propria region of 5–7 villi (from base to apical tip) was analyzed with the Angiotool software (version 0.6a). ERG+ and Ki67+ cell counts were quantified manually in the lamina propria region from 7–8 villi. The proliferating EC% was quantified as a percentage of ERG+/Ki67+ cells among ERG+ cells. The EC# was normalized to the size of the lamina propria region. DAPI+ cell counts and thus the EC% were not quantified, because DAPI staining in intestinal whole-mount samples is too dense to reliably quantify individual nuclei.

For the ear skin analysis, two to three ROIs (850.19 μm x 850.19 μm or 425.10 μm x 425.10 μm) were imaged from the peripheral margin of the ear pinnae, encompassing both inner and outer dermal layers. For the analysis of the ear skin BV area%, lymphatic vessel (LV) areas were first manually removed from PDXL-stained images based on their distinct morphology. The LV area% was quantified as a percentage of the LYVE1+ LV area within the ROI.

For the retinal analysis, five randomly selected ROIs (1024 μm x 1024 μm or 2048 μm x 2048 μm) were imaged from the mid-peripheral zones of the retina for each mouse for the deep plexus BV area% quantifications. Because the superficial plexus has a lower vessel density, 3-5 ROIs (1024 μm x 1024 μm) were imaged per mouse for the superficial plexus BV area% analyses, selecting the ROIs to include areas where the major vessels were.

### Western blotting

Equal amounts of cleared lysates with 25 μg protein from the livers were separated in Novex WedgeWell 4–20% TRIS-Glycine gel 1.0 mm × 15 well (Invitrogen, XP04205BOX). After blotting to polyvinylidene fluoride membranes (Immobilon- FL PVDF, Merck Millipore, IPFL00010), the proteins were detected using rat anti-mouse CD144 (1:1000, 555289, BD Biosciences, RRID: AB_395707) and mouse anti-HSC70 (1:5000, Santa Cruz Biotechnology, sc-7298, RRID: AB_627761) primary antibodies. The blots were then probed with HRP-labeled rabbit anti-rat secondary antibody (1:5000, Abcam, ab6734, RRID: AB_955450), and the signal was visualized with SuperSignal West Pico Chemiluminescent Substrate (Pierce, 34079) or SuperSignal West Femto Maximum Sensitivity Substrate (Pierce, 34096). HSC70 was probed using IRDye 680RD donkey anti-Mouse IgG (1:10 000, LICORbio, 926-68072, RRID: AB_10953628) and was detected using LI-COR Odyssey Fc (LICORbio). Densitometric analysis of the blots was performed using ImageStudio Lite (Version 5.2.5, LICORbio). The values were normalized to HSC70 for protein loading. The uncropped blots are presented in Supplementary Fig. 6.

### Perfusion experiment

Mice were anesthetized with a mixture of xylazine (9 mg/kg) and ketamine (70 mg/kg). The tracer dye *Griffonia (Bandeiraea) Simplicifolia* Lectin I (GSL I, BSL I), Rhodamine, referred to as rhodamine-lectin (8 mg/kg in PBS, Vector laboratories, RL-1102-2) was administered as an intravenous injection through the retro-orbital cavity at a speed of 10 µL/s using a nanoliter pump (Harvard Apparatus Pump 11 Elite (Dual Prog)) and 10 min after the administration, mice were perfused with PBS (11 mL/min for 1 min) to remove the tracer from the bloodstream, followed by perfusion with 4% PFA to fix the tracer. Collected samples were used for immunostainings performed as described above.

### Reporting summary

Further information on research design is available in the Nature Portfolio Reporting Summary linked to this article.

## Supplementary information

Supplementary Information

Description of Additional Supplementary files

Supplementary data 1

Reporting Summary

## Data Availability

Source data for the figures and supplemental figures are provided as a Supplementary Data 1 file. Source data are provided with this paper.
