# Supplementary material for: High-fat diet feeding induces organ-specific vascular remodeling with distinct temporal dynamics in male mice
Source: Commun Biol. 2026 May 9;9:888. doi: 10.1038/s42003-026-10092-4 (PMC13328665; doi:10.1038/s42003-026-10092-4)
Supplement: Supplementary file 1 — Supplementary Information [file 42003_2026_10092_MOESM1_ESM.pdf]

Supplementary materials for:

High-fat diet feeding induces organ-specific vascular remodeling with distinct temporal dynamics in male mice

Emmi Pakarinen[1], Satu Paavonsalo[1], Madeleine H. Lackman[1], Yelin Subashi[1], Hanna M. Ruddock[1], and Sinem Karaman[1,2]

[1]Individualized Drug Therapy Research Program, Faculty of Medicine, University of Helsinki, Helsinki 00014, Finland
[2]Wihuri Research Institute, Helsinki, Finland

Running title: Vascular remodeling in high-fat feeding

Key words: Organotypic vasculature, capillary rarefaction, obesity, weight loss

Correspondence:
Dr. Sinem Karaman
Individualized Drug Therapy Research Program,
Faculty of Medicine, University of Helsinki,
and Wihuri Research Institute
Helsinki 00014, Finland
Phone: +358294125526
Email: sinem.karaman@helsinki.fi

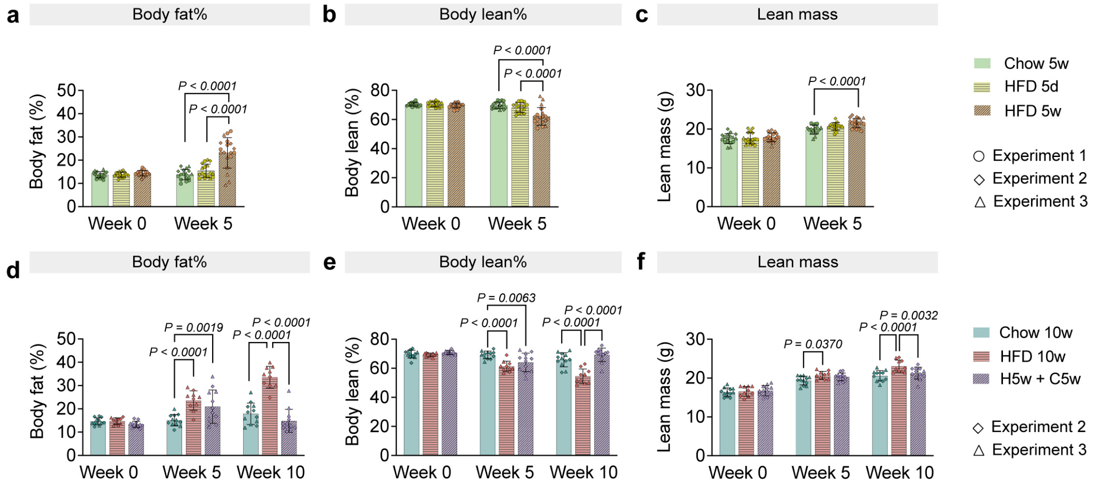

Supplementary figure 1. High-fat diet (HFD) feeding for 5 and 10 weeks increases the body fat percentage and lean mass in mice. Body fat percentages of mice measured a) in the beginning and end of the short-term HFD feeding model and d) in the beginning, midpoint, and end of the long-term HFD feeding model. Lean percentages of mice measured b) in the beginning and end of the short-term HFD feeding model and e) in the beginning, midpoint, and end of the long-term HFD feeding model. Lean masses of mice measured c) in the beginning and end of the short-term HFD feeding model and f) in the beginning, midpoint, and end of the long-term HFD feeding model. In the graphs, each dot represents one mouse, and individual experiments are indicated by different shapes. The number of mice in the graphs, pooled from three independent experiments for the short-term HFD model and from two independent experiments for the long-term HFD model, are the following: Chow 5w (n=17), HFD 5d (n=17), HFD 5w (n=19), Chow 10w (n=12), HFD 10w (n=10), and H5w + C5w (n=12). Error bars represent mean ± standard deviation. Statistical significance was assessed using repeated measures two-way ANOVA followed by Tukey's multiple comparisons test for all graphs a-f. C = Chow, H/HFD = High-fat diet.

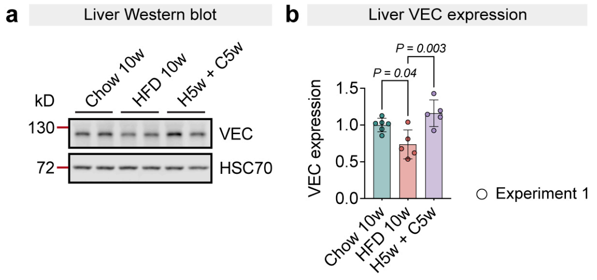

Supplementary figure 2. Reduced VE-Cadherin (VEC) expression in the liver after 10 weeks of high-fat diet (HFD) feeding. a) Western blots and b) related quantifications of VEC normalized to HSC70 in liver lysates in the long-term HFD model. In the graphs, each dot represents one mouse. The number of mice in the graphs are the following: Chow 10w (n=6), HFD 10w (n=5), and H5w + C5w (n=5). Error bars represent mean ± standard deviation. Statistical significance was determined by one-way ANOVA followed by Tukey's multiple comparisons test for graph b. C = Chow, H/HFD = High-fat diet, VEC = VE-Cadherin.

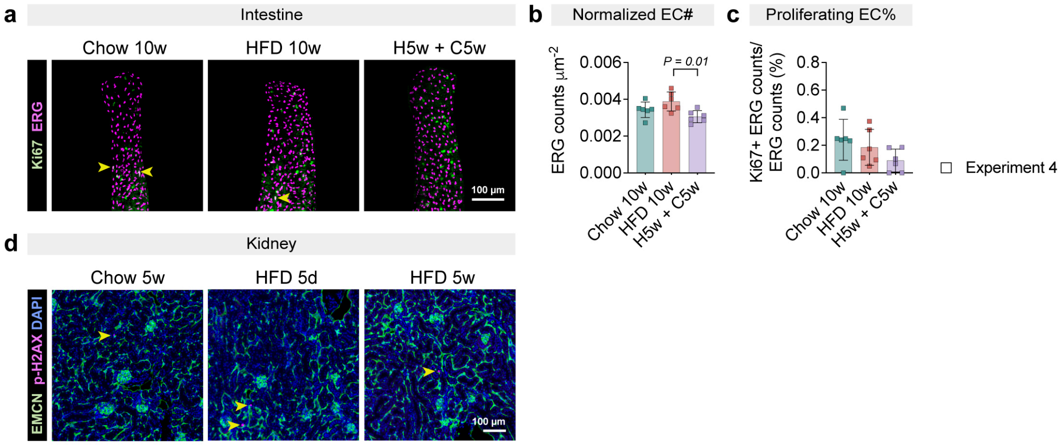

Supplementary figure 3. High-fat diet (HFD) feeding does not increase the proliferation of endothelial cells (ECs) in the intestinal villus or DNA fragmentation in the kidney. a) Representative images of intestine whole-mounts stained for proliferation marker Ki67 (green) and EC marker ERG (magenta), and related quantifications for b) normalized EC# and c) proliferating EC% in the long-term HFD model. The normalized EC# is expressed as the number of ERG+ cells within the lamina propria area and the proliferating EC% is expressed as the % of Ki67+/ERG+ double-positive cells among all ERG+ cells. d) Representative images of blood vessel (EMCN, green), DNA damage marker phosphorylated histone 2 AX (p-H2AX, magenta), and cell nuclei (DAPI, blue) stainings of kidney paraffin sections in the short-term HFD model. Yellow arrows indicate p-H2AX+ cells. In the graphs, each dot represents the mean value of one mouse. Number of mice per group, n=6. Error bars represent mean ± standard deviation. Statistical significance was determined by one-way ANOVA followed by Tukey's multiple comparisons test for graph b. C = Chow, EC = Endothelial cell, EMCN = Endomucin, H/HFD = High-fat diet.

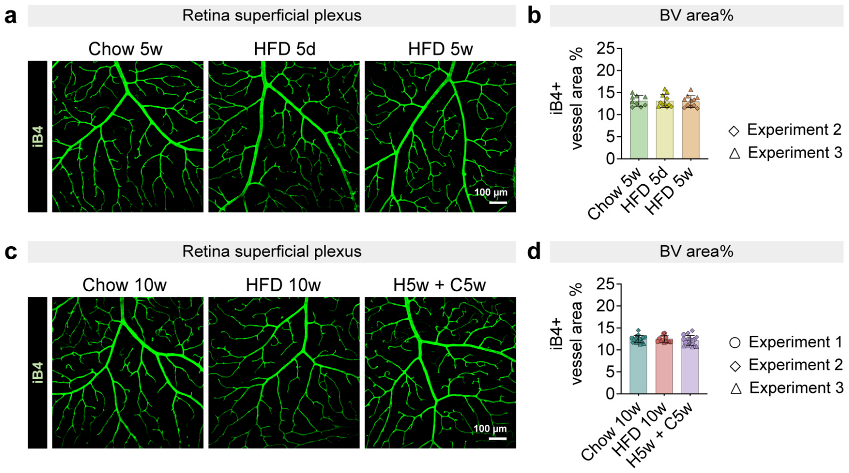

Supplementary figure 4. Blood vessel (BV) coverage does not change in the retinal superficial plexus after high-fat diet (HFD) feeding. a) Representative images of BV (iB4, green) stainings of retinal superficial plexus whole-mounts in the short-term HFD model and related quantifications for b) BV area%. c) Representative images of BV (iB4, green) stainings of retinal superficial plexus whole-mounts in the long-term HFD model and related quantifications for d) BV area%. The BV area% is expressed as the % of iB4+ BV area within the region of interest (ROI). In the graphs, each dot represents the mean value of one mouse, and individual experiments are indicated by different shapes. The number of mice in the graphs, pooled from two independent experiments for the short-term HFD model and from three independent experiments for the long-term HFD model, are the following: Chow 5w (n=9), HFD 5d (n=11), HFD 5w (n=11), Chow 10w (n=18), HFD 10w (n=15), and H5w + C5w (n=17). Error bars represent mean ± standard deviation. BV = Blood vessel, C = Chow, H/HFD = High-fat diet, iB4 = Isolectin B4.

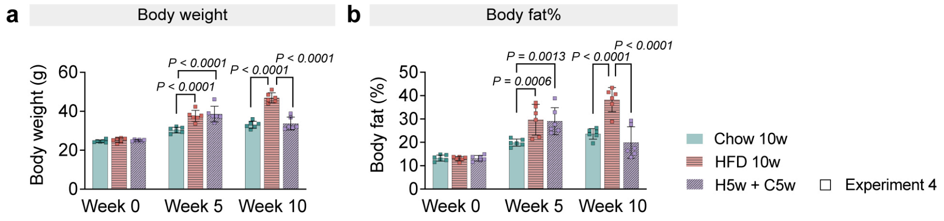

Supplementary figure 5. High-fat diet (HFD) feeding for 5 and 10 weeks increases the body weight and body fat percentage in mice. a) Body weights and b) body fat percentages of mice measured in the beginning, midpoint, and end of the long-term HFD feeding model. In the graphs, each dot represents one mouse. Number of mice per group, n=6. Error bars represent mean ± standard deviation. The repeated measures of two-way ANOVA and Tukey's multiple comparisons test were performed to analyze differences between the groups in graphs a and b. C = Chow, H/HFD = High-fat diet.

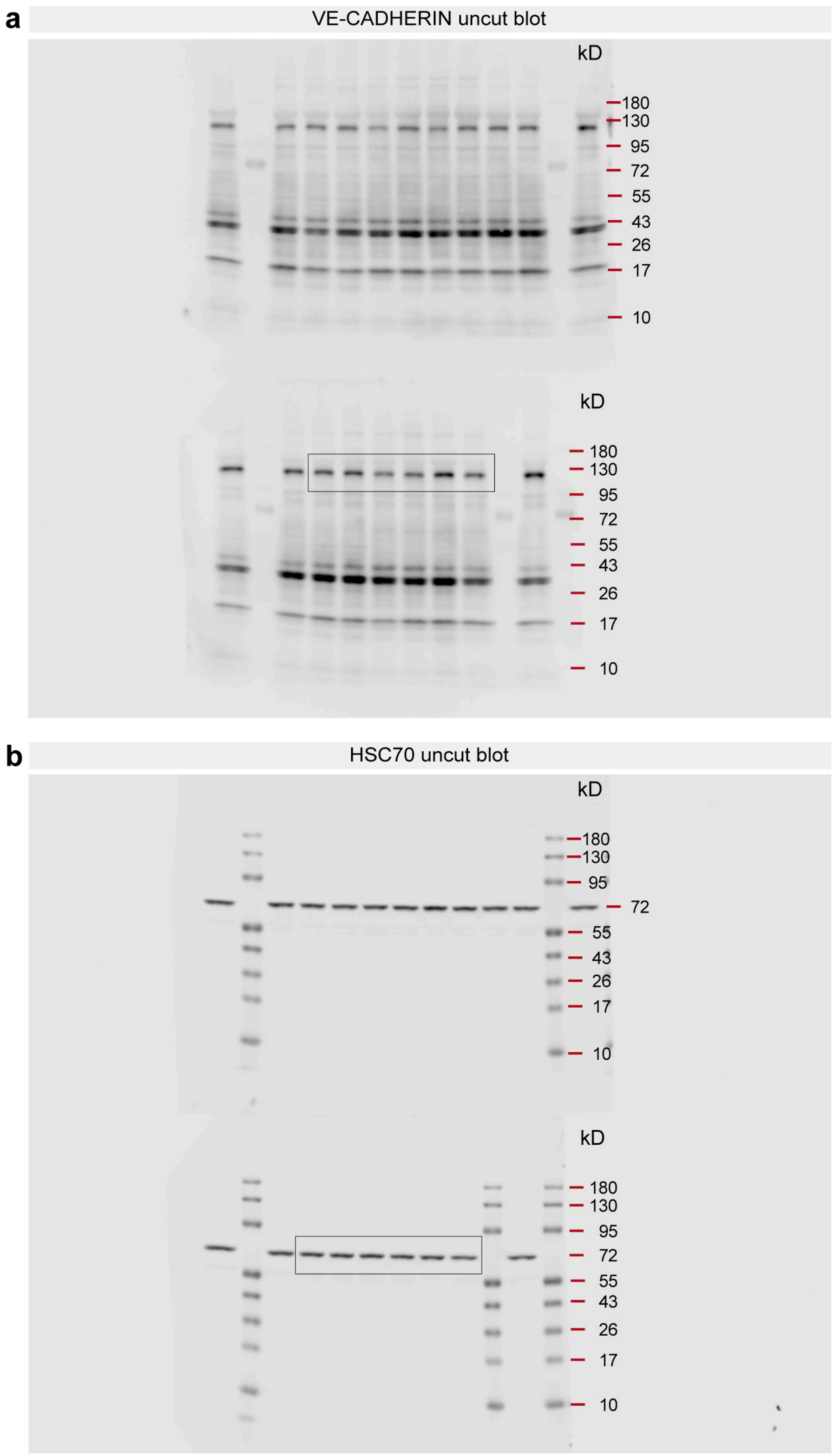

Supplementary figure 6. Uncropped Western blots for Supplementary figure 2. a) Western blots probed with VE-Cadherin antibody. b) Western blots probed with HSC70 antibody. The lanes marked in boxes of uncut blots show the presented lanes in Supplementary figure 2.
